# Of macrophages and red blood cells; a complex love story

**DOI:** 10.3389/fphys.2014.00009

**Published:** 2014-01-30

**Authors:** Djuna Z. de Back, Elena B. Kostova, Marian van Kraaij, Timo K. van den Berg, Robin van Bruggen

**Affiliations:** Landsteiner Laboratory, Department of Blood Cell Research, Academic Medical Center, Sanquin Research, University of AmsterdamAmsterdam, Netherlands

**Keywords:** red blood cell, macrophages, spleen, erythropoiesis, phagocytosis

## Abstract

Macrophages tightly control the production and clearance of red blood cells (RBC). During steady state hematopoiesis, approximately 10^10^ RBC are produced per hour within erythroblastic islands in humans. In these erythroblastic islands, resident bone marrow macrophages provide erythroblasts with interactions that are essential for erythroid development. New evidence suggests that not only under homeostasis but also under stress conditions, macrophages play an important role in promoting erythropoiesis. Once RBC have matured, these cells remain in circulation for about 120 days. At the end of their life span, RBC are cleared by macrophages residing in the spleen and the liver. Current theories about the removal of senescent RBC and the essential role of macrophages will be discussed as well as the role of macrophages in facilitating the removal of damaged cellular content from the RBC. In this review we will provide an overview on the role of macrophages in the regulation of RBC production, maintenance and clearance. In addition, we will discuss the interactions between these two cell types during transfer of immune complexes and pathogens from RBC to macrophages.

## From erythroblastic islands to clearance

During their development and mature life, red blood cells (RBC) interact numerous times with macrophages, first during their development in the bone marrow, later in the blood stream with macrophages in the liver and spleen. All of these interactions are essential for efficient production under different conditions, to maintain RBC homeostasis or to ensure the correct removal of aged or damaged RBC. In this review, an overview of the different processes in which RBC-macrophage interactions play an important role is given.

## The erythroblastic island; a lying-inroom for effective erythropoiesis

Adult erythropoiesis is a tightly regulated process which occurs in the bone marrow. It consists of several developmental stages: hematopoietic stem cell, burst- forming unit- erythroid (BFU-E), colony- forming unit- erythroid (CFU-E), proerythroblast, basophilic erythroblast, polychromatic erythroblast, orthochromatic erythroblast, reticulocyte and ultimately to mature RBC (Manwani and Bieker, 2008; An and Mohandas, 2011). Erythrocyte production is regulated by a negative feedback loop where oxygen levels determine plasma levels of erythropoietin (Epo). Even though there are a number of growth factors known to participate in the regulation of erythropoiesis, Epo has been identified as the master regulator of RBC production (Ji et al., 2011). Epo drives RBC precursor proliferation and differentiation and can prevent erythroblast apoptosis (Koury and Bondurant, 1990). Furthermore, terminal erythropoiesis has been reported to take place in a specialized microenvironment called the erythroblastic island. Erythroblastic islands were first described in 1958 by Besis who characterized them by analysing transmission electron micrographs of bone marrow sections. He showed a structure containing a macrophage surrounded by developing erythroblasts (Bessis, 1958) and concluded that macrophages actively participate in erythroid development by providing iron for heme synthesis and by phagocytosing expelled nuclei during final erythroid differentiation (Bessis and Breton-Gorius, 1962). Moreover, in 1972 a functional role of erythroblastic islands was demonstrated by comparing erythroblastic islands of normal rats and hypertransfused rats and showing that hypertransfused rats exhibit a significant reduction in erythroblast islands numbers by using 3D electron microscopy (Mohandas and Prenant, 1978). This finding suggests that suppression of erythropoiesis by means of RBC transfusion would result in diminished erythroid island formation, linking erythropoiesis rate to the number of erythroblastic islands. Erythroblastic islands have been described during primitive erythropoiesis as well. Even though erythroblasts in the yolk sac do not require a specialized microenvironment for development, they attach closely to structures highly similar to erythroblastic islands (McGrath et al., 2008). Moreover, erythroblastic islands have been reported in other sites for definitive erythropoiesis such as fetal liver and splenic red pulp (Manwani and Bieker, 2008).

Structurally, unlike megakaryocytes which are situated close to bone marrow sinusoids, to ensure fast egress into circulation when platelets are needed, erythroblastic islands are unevenly distributed inside the marrow, with islands adjacent or distant from the sinusoids. However, it should be noted that *in vivo* studies on erythroblastic islands in humans is virtually impossible, therefore a lot of experiments have been performed using animal models. In a study dissecting rat bone marrow, quantitative light and electron microscopy analysis shows that nonadjacent islands accommodate more pro-erythroblasts, while on the other hand islands situated next to sinusoids contain more differentiated erythroblasts (Yokoyama et al., 2003). This interesting observation proposes that erythroblastic islands are capable of migrating towards bone marrow sinusoids as erythroid precursors mature. It is possible that interactions between erythroblast and central macrophage trigger a cascade leading to the release of macrophage proteases, which would aid extracellular matrix remodeling, and hence island progression to the sinusoid. Moreover, erythroblasts can potentially attach and detach from one central macrophage to another, thus facilitating their movement further to sinusoids. Nevertheless, the interaction between macrophage and differentiating erythroid precursors appear to be essential throughout erythropoiesis.

## Role of macrophages in erythropoiesis

Despite the fact that erythroblastic islands were described a few decades ago, understanding of the interactions between macrophages and erythroblasts during erythropoiesis is incomplete. To begin with, the specific erythroblast island cellular composition can vary depending on the species. Evidence obtained from tissue sections of rat femur shows roughly 10 erythroblasts per island (Yokoyama et al., 2002), while islands collected from human bone marrow can contain 5–30 erythroblasts surrounding a central macrophage (Lee et al., 1988).

**Figure 1:**
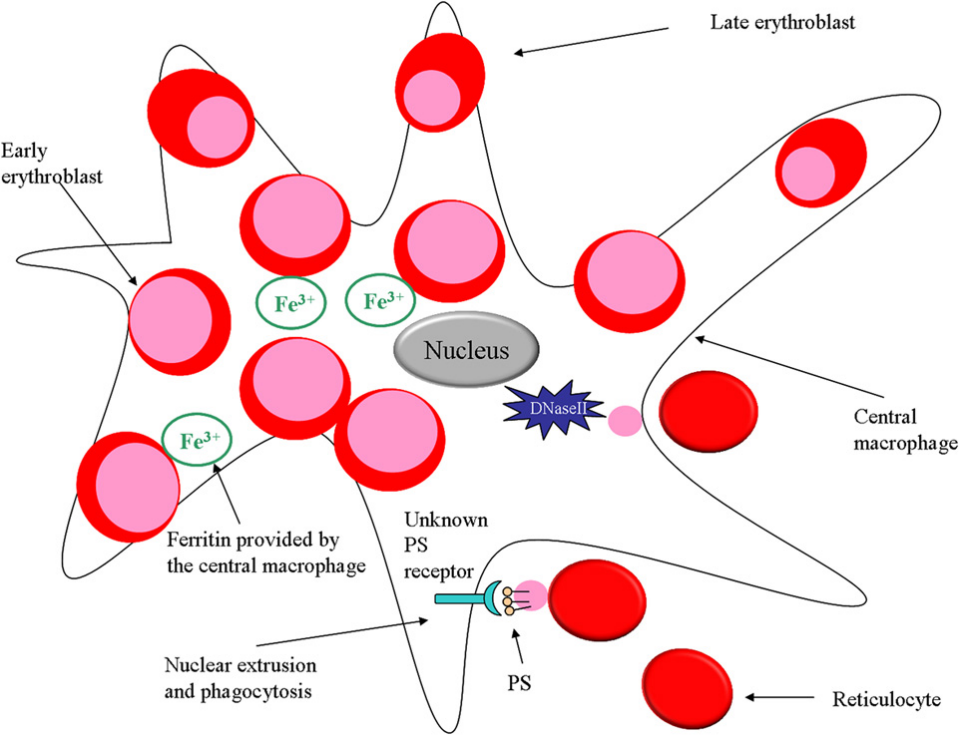
**Role of macrophages in erythropoiesis**. In the erythroid niche, macrophages not only provide iron for developing erythroblasts but also phagocytose expelled nuclei. Furthermore, the macrophage protein DNaseII is important for breakdown of nuclei that are expelled by erythroblasts.

As mentioned earlier, macrophages were proposed to promote erythropoiesis by directly transferring iron to erythroid progenitors (Bessis and Breton-Gorius, 1962). It should be noted that splenic red pulp macrophages are mainly responsible for iron return to bone marrow from recycling of senescent and damaged erythrocytes, after catabolism of hemoglobin molecules. Recently it was demonstrated in an erythroblastic island culture that ferritin produced by macrophages is released by exocytosis and engulfed by erythroblasts via endocytosis (Figure 1). Once inside the erythroblast, iron is released from ferritin upon acidification and proteolysis, thus being subsequently available for heme production in the erythroid precursor cell (Dautry-Varsat et al., 1983; Leimberg et al., 2008; Hentze et al., 2010; Li et al., 2010b).

There are several examples in literature showing that macrophages not only promote erythropoiesis by providing iron, but also by directly stimulating proliferation and survival of erythroblasts. When erythroblasts are cultured *in vitro*, the erythroid precursors attached to macrophages are subjected to enhanced proliferation compared to non-attached erythroblasts proposing that macrophages may augment the response to Epo upon direct interaction with erythroblasts (Rhodes et al., 2008).

It has been demonstrated that abnormal macrophage differentiation can have a direct effect on erythroblastic island function. For instance, when retinoblastoma tumor suppressor (Rb) protein is knocked out in a mouse model, fetuses die *in utero* due to anemia (Clarke et al., 1992; Jacks et al., 1992; Lee et al., 1992). Rb is a nuclear factor that regulates cell cycle transition from G1 to S phase and is critical for macrophage differentiation (Iavarone et al., 2004).

Cytoskeletal-associated protein palladin has also been implicated in macrophage function. It is a protein that localizes in focal adhesions of stress fibers together with α- actinin, thus promoting cytoskeletal dynamic rearrangements and adherence to the extracellular matrix. Knocking out palladin in a mouse model is embryonic lethal due to anemia caused by erythroblast cell death and aberrant terminal erythroid differentiation. Fetal liver erythroblastic island integrity is compromised and *in vitro* erythroblastic island formation is perturbed in palladin^−/−^ mice due to an intrinsic macrophage defect (Liu et al., 2007).

In addition, the macrophage transcription factor c-Maf has been identified as a critical component in definitive erythropoiesis in fetal liver. Deletion of c-Maf leads to severe erythropenia *in utero* and significant reduction in fetal liver erythroblastic island formation compared to wild type. The observed defective erythropoiesis seems to be due to an abnormal erythroid niche and not to a cell autonomous effect (Kusakabe et al., 2011). These examples clearly show that macrophages are crucial participants in erythroid development as targeted deletion of enzymes, proteins and transcription factors responsible for macrophage proliferation and survival ultimately results in perturbed erythroid niche formation and defective erythropoiesis.

## Direct interactions between erythroblasts and macrophages within erythroblastic islands

**Figure 2:**
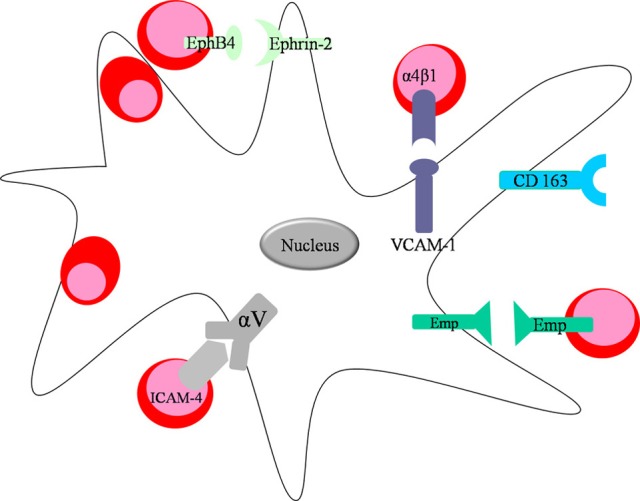
**Direct interactions between the central macrophage and developing erythroblasts**. Macrophages express VCAM-1 and integrin αV on their surface facilitating interaction with erythroblasts through integrin α4β 1 and ICAM-4, respectively. Moreover, both erythroid cells and macrophages express Emp on their surface promoting their interactions in the niche. Central macrophage and erythroblast can also interact via the ligand-receptor pair EphB4 (expressed on the surface of erythroblasts) and Ephrin-2, found on macrophages.

Needless to say, the function and integrity of erythroblastic islands is tightly related to the molecular interactions occurring between erythroid precursors and the central macrophage. Erythroblasts express a myriad of adhesion molecules throughout their differentiation, which not only facilitate adhesion to extracellular matrix proteins such as fibronectin and laminin, but also attachment to the central macrophage. The first molecule identified on the surface of both central macrophages and erythroblasts is Erythroblast macrophage protein (Emp), a protein that promotes binding between the two cell types (Hanspal and Hanspal, 1994) (Figure 2). In erythroblastic island cultures absence of Emp leads to aberrant erythropoiesis and increased levels of apoptosis (Hanspal et al., 1998), suggesting that the direct association between the central macrophage and the erythroblasts is essential for erythroid maturation and prevention of cell death. In support of those findings are the *in vivo* experiments performed with Emp deficient mice, which show that those animals die before birth owing to severe anemia (Soni et al., 2006). Next, another important molecular interaction found in erythroblastic islands occurs between the α4β 1 integrin (Very Late Antigen 4; VLA-4) on erythroblasts and vascular cell adhesion molecule 1 (VCAM-1) on central macrophages (Figure 2). The biological significance of this receptor pair is underlined in experiments in which erythroid island formation is perturbed by antibodies against α4β 1 integrin or VCAM-1 (Sadahira et al., 1995). Studies in mice have shown that integrins have a pivotal role in stress erythropoiesis (Ulyanova et al., 2011). In addition, intercellular adhesion molecule 4 (ICAM-4) expressed on erythroblasts and αV integrin present on macrophages (Figure 2) have a vital function in maintaining island integrity, since disrupting the binding between the two molecules using synthetic peptides leads to a diminished number of erythroblastic islands. In an *in vivo* model, utilizing ICAM-4 deficient mice, reduced island formation is observed as well (Lee et al., 2006). Moreover, a secreted form of ICAM-4 possibly regulates terminal erythropoiesis by competing with surface ICAM-4 for αV integrin on central macrophages preventing the interaction between erythroblasts and macrophage (Lee et al., 2003).

CD163 is another macrophage receptor that interacts with erythroblasts (Fabriek et al., 2007) (Figure 2). CD163 is well-known to scavenge hemoglobin-haptoglobin complexes, thus clearing free hemoglobin from circulation (Kristiansen et al., 2001). CD163 contains an erythroblast adhesion motif as well, which mediates binding of macrophages to erythroid precursors facilitating erythroblast expansion and survival (Fabriek et al., 2007). Future studies are required to further characterize the contribution of the direct interactions between erythroblasts and macrophages on island structural integrity and on signaling pathways during erythropoiesis. For instance, the specific receptor/ligand pairs on erythroblasts and macrophage that are involved in cell-cycle regulation during erythropoiesis have not yet been identified. Likely candidates are macrophage membrane protein Ephrin-2 (HTK ligand) binding erythroid receptor EphB4 (HTK) (Figure 2) (Inada et al., 1997; Suenobu et al., 2002) and c-kit ligand interacting with c-kit on erythroblasts (Muta et al., 1995). Both macrophage surface molecules have been shown to augment erythroid proliferation.

## Soluble factors important in erythroblastic islands

**Figure 3:**
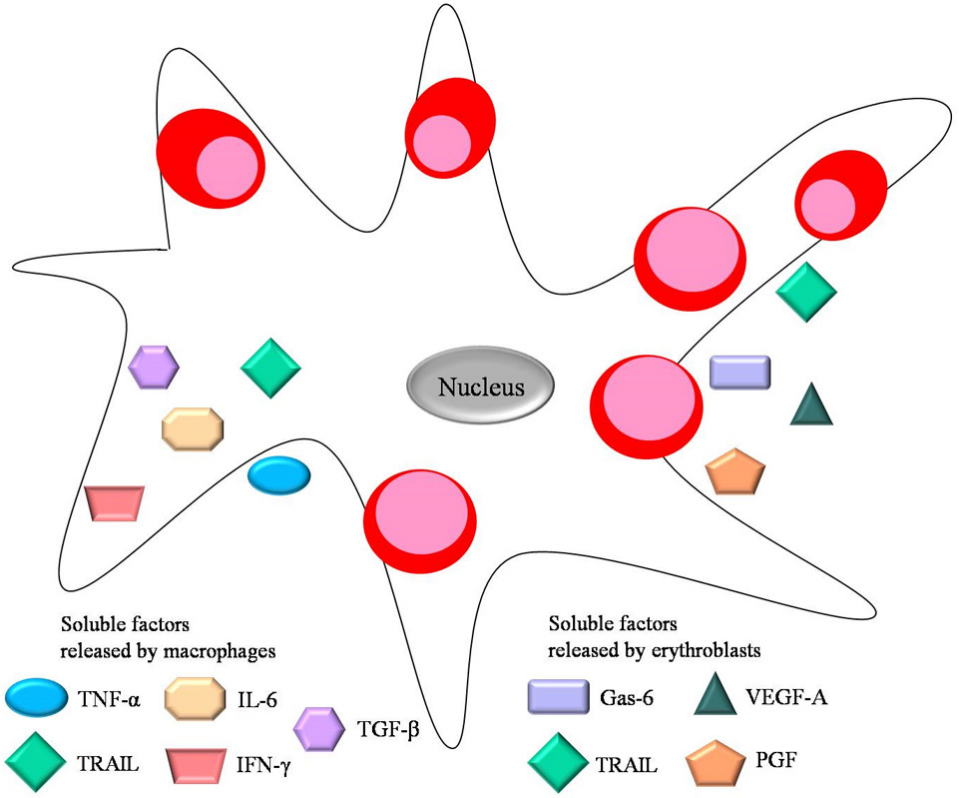
**Soluble factors important for erythropoiesis**. Both central macrophage and erythroblast secrete soluble factors during erythropiesis. These include the negative regulators TNF-α, TGF-β, IL-6, IFN-γ released by the central macrophage, and the positive regulators Gas-6, VEGF-A and PGF released by erythroblasts. TRAIL is a negative regulator secreted by both erythroid cells and macrophages.

Interestingly, there is experimental evidence suggesting that erythroblasts might modulate island integrity by secreting angiogenic factors such as vascular endothelial growth factor A (VEGF-A) and placental growth factor (PGF) (Tordjman et al., 2001) (Figure 3). One can hypothesize that release of these molecules can contribute to the reticulocytes' egression into the vasculature by regulating the stability of endothelial junctions. Moreover, even though erythroblasts do not express receptors for VEGF-A and PGF on their surface, central macrophages do, suggesting that erythroblasts may secrete those factors as paracrine modulators of macrophage proliferation and survival in the erythroid niche.

On the other hand, growth arrest-specific 6 (Gas-6) released by erythroblasts has been proposed to modulate the erythroid microenvironment during erythropoiesis. Gas-6 is a molecule normally associated with proliferation and survival of non-erythroid cells. However, erythroblasts can secrete Gas-6 in response to Epo, thus positively regulating Epo signaling through phosphoinositide 3 kinase (PI3K) and Akt activation (Angelillo-Scherrer et al., 2008).

Furthermore, there are several soluble factors secreted by macrophages within the erythroblastic island that negatively regulate erythropoiesis. These include cytokines, chemokines and interleukins including interleukin 6 (IL-6), transforming growth factor-β (TGF-β), tumor necrosis factor-α (TNF-α), and interferon-γ (INF-γ), all of which are associated with chronic inflammation and tumor progression. For instance, patients suffering from chronic inflammation have high levels of inflammatory cytokines in the bone marrow which inhibit erythropoiesis (Means, 2004). The mechanism by which TNF-α released by macrophages suppresses erythropoiesis involves caspase-mediated cleavage of GATA-1, a pivotal transcriptional regulator of erythroid development. This leads to apoptosis (De Maria et al., 1999) or delayed proliferation (Dai et al., 2003) of the erythroid progenitors. During terminal differentiation GATA-1 is normally protected from caspase cleavage by heat shock protein 70 (Hsp-70) (Ribeil et al., 2007). Thus Hsp-70 can regulate erythropoiesis by preventing the induction of apoptosis which would negatively influence RBC production. Furthermore, secreted TNF-α can trigger release of metalloproteases by macrophages in order to remodel extracellular matrix in other tissues, however a similar event occurring in the context of erythroblastic islands would have deleterious effects on the microenvironment's integrity.

Bone marrow macrophages isolated from myelodysplastic syndrome (MDS) patients release higher levels of TNF-α compared to macrophages from healthy donors (Flores-Figueroa et al., 2002). Moreover, they present with an increased apoptotic index, suggesting an abnormal macrophage function inside the erythroid niche in MDS.

Additionally, TGF-β released by macrophages is known to block erythroblast proliferation and survival via a mechanism different than apoptosis, and at the same time enhances erythroid differentiation (Zermati et al., 2000). TGF-β can activate Rho and Rac GTPases, influencing cell cytoskeletal stability and organization in various cell types (Maddala et al., 2003), including erythroblasts, which require stable cytoskeletal integrity for normal development. Increased levels of the inflammatory cytokine IFN-γ can lead to secretion of TNF-related apoptosis inducing ligand (TRAIL), by both macrophages and erythroblasts (Zamai et al., 2000). TRAIL blocks erythroid differentiation by a activating the ERK/MAPK (extracellular signal-regulated kinase/mitogen-activated protein kinase) pathway (Secchiero et al., 2004). All soluble factors discussed above are depicted in Figure 3. Notably, many studies on erythroblastic islands and erythroid proliferation and survival manage to establish the crucial link between macrophage and erythroblast interactions and their effects on erythroid niche development.

## Macrophages phagocytose nuclei expelled from erythroblasts

During the final stage of terminal erythroid differentiation, the erythroblast expels its nucleus as part of its maturation into a reticulocyte. The macrophage has a critical role during this process since it will phagocytose the expelled nucleus, aiding erythropoiesis (Seki and Shirasawa, 1965; Skutelsky and Danon, 1972). Both the macrophage and the erythroblast/reticulocyte are equipped with adhesion molecules promoting the retention of the nucleus on the surface of the macrophage before phagocytosis takes place. It has been shown that Emp (Soni et al., 2006) and β 1 integrin (Lee et al., 2004) predominantly distribute on the nucleus after expulsion, thus maintaining the interaction between the nucleus and the macrophage. Moreover, studies performed with fetal liver erythroblasts demonstrate that expelled nuclei expose phosphatidylserine (PS) on their surface for 10 min after expulsion (Yoshida et al., 2005) (Figure 1). This observation corroborates with the finding that the time frame between nucleus expulsion and phagocytosis is 10 min (Allen and Testa, 1991), which suggests that PS might be assisting in the adhesion of the nucleus to the macrophage prior to phagocytosis. PS is a membrane component normally situated on the inner leaflet of the cell membrane. An ATP-dependent aminophospholipid translocase enzyme maintains the lipid asymmetry by keeping PS on the inside of the plasma membrane. PS exposure on the cell surface is considered an apoptotic signal targeting cells undergoing cell death for clearance by phagocytes expressing PS receptors. Moreover, PS externalization can be a direct effect of ATP depletion in the cell. In addition, Yoshida and colleagues have demonstrated that expelled nuclei expose PS and lack ATP (Yoshida et al., 2005). Furthermore, the PS-binding protein lactadherin (also known as MFGE8) which normally serves as a bridging molecule between an apoptotic cell and a phagocyte (Hanayama et al., 2002), has been shown to be crucial for phagocytosis of extruded erythroblast nucleus as well. A mutated form of lactadherin, not being able to bind PS, inhibits phagocytosis of expelled nuclei (Yoshida et al., 2005).

Another study that suggests an important role for macrophages for the phagocytosis and degradation of expelled nuclei during erythropoiesis made use of DNase II deficient mice. DNase II is an enzyme necessary to degrade nuclear DNA after phagocytosis and proves to be essential for erythropoiesis (Kawane et al., 2001) (Figure 1). It was shown that fetal liver macrophages from DNase II deficient mice are unable to degrade the ingested nuclei and that DNase II-null mice die *in utero* due to severe anemia.

## Macrophages in erythropoiesis: in health and disease

Despite the fact that our knowledge of macrophage-erythroblast interactions in erythroblastic islands and their role in erythroid development is expanding, it is important to realize that many of the experiments have been conducted *in vitro*. In a recent paper Chow and colleagues elegantly show that CD169^+^ macrophages promote erythropoiesis in steady state and under stress *in vivo* (Chow et al., 2013). CD169 was first described to be a marker of central macrophages in the erythroblastic island more than two decades ago (Crocker et al., 1990). In the study by Chow et al. depletion of CD169^+^ macrophages leads to a decreased number of erythroblasts in the bone marrow and mild iron-deficiency anemia. Furthermore, CD169^+^ macrophages appear to be essential for recovery from hemolytic anemia, acute blood loss and myeloablation. On the other hand, macrophage depletion can rescue the phenotype of polycythemia vera in a JAK2^V617F^- driven mouse model. These findings suggest that macrophages are not only a critical component during erythroid maturation in steady state, but also during stress and disease.

To support the speculation that macrophages might also have a function in erythropoiesis in the context of disease and to further characterize their importance in erythropoiesis *in vivo*, Ramos and colleagues show that macrophages regulate erythroid development in polycythemia vera, β-thalassemia and anemia (Ramos et al., 2013). Chemical depletion of macrophages by clodronate liposome administration prevents mice from recovering from induced anemia, suggesting an essential function of macrophages in promoting stress erythropoiesis *in vivo*. Conversely, macrophage depletion not only improves the phenotype of polycythemia vera and reverses the pathological aspects of the disease, but also alleviates anemia caused by β-thalassemia. These results propose an important dual role of macrophages in physiological and pathological erythropoiesis *in vivo*. Both studies suggest that macrophages exert two seemingly contradictory actions on erythropoiesis. On one hand, macrophages are indispensable for stress erythropoiesis *in vivo*. In their absence erythroid production in the bone marrow and spleen in response to bleeding is impaired. However, macrophages can also be deleterious in the context of polycythemia vera and β-thalassemia, since depletion of macrophages leads to a decreased disease pathology. Moreover, *ex vivo* cultured human macrophages from polycythemia vera patients promote proliferation of human erythroblasts and diminish differentiation. This suggests a function for macrophages in disease progression since polycythemia vera is characterized by an overactive erythron and excessive erythropoiesis (Ramos et al., 2013). These findings might pave the way to future therapies implementing macrophage depletion in the treatment of erythroid disorders like polycythemia vera and β-thalassemia. These and other studies demonstrate the importance of erythroblastic islands and more specifically, the interaction between macrophage and erythroblasts for RBC maturation in physiological and pathological conditions. Future experiments are necessary to examine in more detail the involvement of macrophages in red blood cell production in steady state and disease. Even though, animal models are necessary to illustrate the *in vivo* situation, one should take these studies into consideration with caution. It should be noted that erythroid development within erythroblastic islands differs between mice and men.

## Interaction of RBC and macrophages in the bloodstream

After they are produced in the bone marrow, RBC remain in circulation for roughly 120 days. Throughout their life span RBC pass the liver and the spleen numerous times where they encounter resident macrophages (Crosby, 1959). The interactions between macrophages and RBC taking place in liver and spleen are important for RBC homeostasis and ultimately for the removal and degradation of aged RBC at the end of their life span (Mebius and Kraal, 2005). In addition, macrophages take up immune complexes and pathogens bound to complement receptor 1 (CR1) on the RBC and can clear intracellular pathogens such as *Plasmodium* from the RBC, leaving the RBC intact and allowing the return of the RBC into circulation (Wilson et al., 1987). The different molecular interactions that are important for these different processes are discussed below.

## The removal of intracellular inclusions by macrophages of the spleen

The macrophages of the spleen have a remarkable function that enables them to remove unwanted damage from the RBC membrane, leaving the RBC intact (Crosby, 1957; Schnitzer et al., 1972). Removal of these intracellular inclusions seems to occur within the open circulation where the RBC are also checked for their loss of deformability to check for age. To achieve this, RBC must pass through the endothelial slits of the sinus to reenter the blood circulation. During this course, cells that are non-deformable will be removed from the circulation by residential macrophages. In the mean-time all inclusion bodies are also being removed. In splenectomized patients or in patients with a non-functional spleen, phagocytosis of the inclusion bodies fails and results in a retention of a variety of intracellular inclusions within the RBC, such as Howell-jolly bodies (inclusions of nuclear chromatin remnants) (Wilkins and Wright, 2000), Heinz bodies (inclusions of denatured hemoglobin caused by oxidative damage) (Wilkins and Wright, 2000) siderocytes (RBC containing granules of iron that are not part of the cell's hemoglobin) (Wilkins and Wright, 2000) and Pappenheimer bodies inclusion bodies formed by phagosomes that have been engulfing excessive amounts of iron (Wilkins and Wright, 2000).

Back in 1957 Crosby already showed that when siderocytes, tagged with radioactive chromium, were injected into a healthy patient with a functional spleen, there was a decline in siderocyte count without the loss of chromium labeled RBC. When the same amount of siderocytes was injected into a splenectomized patient, the amount of siderocytes remained unaltered during the 24-h period of observation. This study hereby demonstrated that passage through the spleen can lead to clearance of damage that is accumulating in the circulating RBC. Furthermore, it revealed that processing of damage from RBC can take place while leaving the RBC intact. Thus, it seems that the spleen and the residential macrophages are highly important in maintaining RBC “healthy.” RBC are of course unable to synthesize new proteins, and although equipped with enzyme systems to counteract the potential toxic effects of the oxygen they transport, they will sustain oxidative damage throughout their life resulting in the formation of Heinz bodies (Harley, 1965). The molecular mechanism that underlies the removal of inclusion bodies is largely unknown. In Willekens et al. (2003) presented an analogy to the removal of Heinz bodies when discussing RBC that lose hemoglobin through vesiculation. Via the process of RBC vesiculation the RBC loses aggregated hemoglobin, which is important to maintain the homeostasis of RBC, increases in density and becomes smaller (Piomelli and Seaman, 1993). It was suggested that this process is also facilitated by the macrophages of the spleen, in which older cells vesiculate more than younger ones. Clearly, macrophages play a pivotal role in the clearance of damaged content from circulating RBC (Crosby, 1957; Willekens et al., 2003) and vesiculation is an interesting and plausible mechanism to explain the efficient removal of damaged content while leaving the RBC intact (Wilson et al., 1987). The molecular mechanism by which macrophages in the spleen would be facilitating RBC vesiculation is still unknown. Ultrastructural studies of spleens from monkeys infected with *Plasmodium knowlesi* suggest that the spleen also removes malaria parasites from red cells, in which once again phagocytes play the main role (Schnitzer et al., 1972). In addition, interesting work by Buffet and colleagues has pointed out that the removal of malaria parasites from RBC occurs in the red pulp of the spleen, using perfused human spleens to proof this point (Buffet et al., 2006). Several studies suggest that due to this function the spleen plays a major protective role in naïve patients (Bachmann et al., 2009; Munasinghe et al., 2009). They all showed that disease severity, parasitemia and mortality were higher in splenectomized patients. This supports the hypothesis that without a functional spleen, there will be no splenic retention which could explain why the outcome in splenectomized patients compared to patients who have a functional spleen is worse.

## RBC removal by macrophages

Residential macrophages of the spleen are able to scrutinize passing RBC and remove those from the circulation that are at the end of their lifespan or have sustained damage beyond repair (Mebius and Kraal, 2005). For example: deformed RBC that have been produced by mistake by the bone marrow or RBC that are affected by hereditary spherocytosis will be taken up by spleen macrophages in the red pulp (Crary and Buchanan, 2009).

At present there is no consensus as to how red pulp macrophages determine which RBC need to be cleared and which can be repaired and/or maintained. It is difficult to identify RBC that are destined to be cleared *in vivo* due to the fact that RBC that are carrying a removal signal will most likely be phagocytosed and hence will no longer be available for analysis. In addition, the proportion of RBC that is daily cleared is a mere 0.8% per day, thus leaving only a very small number of RBC that will carry the removal signals at any given moment a blood sample is taken. Therefore, most of the theories on possible removal signals for RBC phagocytosis are based on *in vitro* work or on data generated in animal models.

The RBC aging phenotype, according to our current knowledge, is associated with a decline in metabolic activity and progressive membrane remodeling, due to for example oxidative stress and vesiculation. Concomitantly, the RBC becomes smaller and more dense (Piomelli and Seaman, 1993). Despite these cumulative events, the removal signals do not seem to gradually accumulate on RBC. On the opposite, they appear as a snap, rapid and non-linear cascade of events at the terminal stage of the aging process, probably shortly before RBC are removed by macrophages (Franco, 2009). Taking into account that RBC are unable to synthesize new proteins, all “removal” markers must derive from modifications in pre-existing molecules or to the acquisition of plasma-derived opsonins directed against these modifications. Although RBC do not undergo classical apoptosis since they do not contain a nucleus, mitochondria or other cellular organelles, the process they undergo has already been termed “eryptosis” since it exhibits many similarities with programmed cell death (Lang et al., 2005). For instance, it is highly likely that phagocytosis of senescent RBC will be non-inflammatory. Thus far, there have been several mechanisms postulated for the clearance of senescent RBC by macrophages.

## Band 3-based clearance mechanism

**Figure 4:**
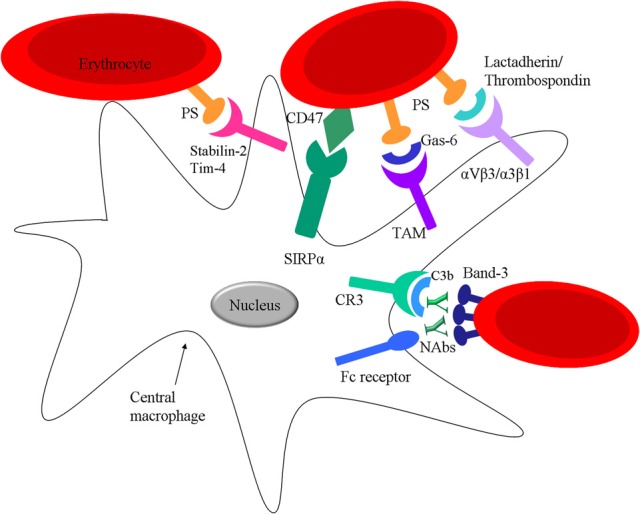
**Interactions between mature RBC and spleen macrophages**. RBC can interact with spleen macrophages via direct receptor ligand pairing or via bridging molecules. Ageing RBC express PS on their surface which can directly bind to Stabilin-2 or Tim-4 on the macrophage or via opsonins such as Gas-6, lactadherin or thrombospondin-1. RBC express CR1 on their surface which can bind C3b osponized particles and further facilitate interaction with spleen macrophages via CR1 and CR3. Nabs can bind Band-3 on the surface of RBC targeting the cell for clearance via Fc receptors on the spleen macrophages. Moreover, RBC express CD47 which binds SIRPα on macrophages.

Band 3, a transmembrane protein that constitutes 25% of the total amount of RBC membrane proteins, has been postulated to be the major target of natural occurring antibodies (NAbs) of the IgG isotype and might be a central step in clearance of senescent and damaged RBC that is mediated by macrophages (Lutz, 2004; Arese et al., 2005; Kay, 2005) Band 3 has two different domains: the membrane spanning domain that catalyses anion exchange and is recognized by Nabs (after clustering) (Figure 4), and a cytoplasmic domain that regulates the structure and function of the RBC by binding different proteins (Zhang et al., 2000; Pantaleo et al., 2008). There is still debate about the mechanism that leads to formation of the epitope on band 3 that results in binding of NAbs. It is thought that oxidative damage to hemoglobin, occurring throughout its lifecycle, and the following formation of hemichromes which bind to band 3, can in time lead to band 3 clustering (Pantaleo et al., 2008; Arashiki et al., 2013). NAbs show an enhanced affinity to band 3 clusters (Low, 1986; Mannu et al., 1995; Hornig and Lutz, 2000). Another hypothesis is that proteolytic degradation of band 3 is essential to shape the band 3 epitopes recognized by NAbs (Kay, 2004).

However, NAbs are not efficient opsonins, due to their low affinity and low circulation numbers. It has been hypothesized that phagocytosis of RBC can be enhanced by the activation of the classical pathway of the complement system after NAb binding. These NAbs preferentially generate C3b_2_–IgG complexes in the presence of active complement (Lutz et al., 1993). Once the classical pathway is activated a significantly lower amount of NAbs is needed for induction of phagocytosis (Lutz et al., 1990). This is because once opsonized with C3b, C3b will form complexes with NAbs that are more resistant to inactivation factors such as: H and I. Red pulp macrophages express CR1 (C3b-receptor, CD35) and CR3 (iC3b-receptor, CD11b/CD18) (Burger and van Bruggen, unpublished) that would enable them to recognize and phagocytose complement opsonized RBC (Figure 4). However, phagocytosis through either of these receptors usually results in the secretion of pro-inflammatory cytokines, although the opposite effects, thus an inhibition of the secretion of pro-inflammatory cytokines has also been shown (Morelli et al., 2003). The effect of complement receptor-mediated phagocytosis on cytokine secretion in macrophages of the spleen is currently under investigation.

## Removal of aged RBC by phosphatidylserine recognizing receptors on macrophages

In healthy cells phosphatidylserine (PS), is normally found on the inner leaflet of the RBC membrane. However, when apoptosis is induced a large increase is seen in the amounts of PS exposed at the cell surface. This increase in PS expression is proposed to be an “eat me” signal for phagocyte recognition of an apoptotic cell, resulting in a non-inflammatory clearance of the dying cell (Fernandez-Boyanapalli et al., 2009).

In an *in vivo* experiment, an increase in PS exposure is seen with RBC age, correlated to the RBC clearance from the circulation (Boas et al., 1998). For a long time it has been proposed that apoptotic cells that express PS can be cleared from circulation via macrophages by recognizing them through specific PS-receptors (Li et al., 2003). Yet recently, various receptors have been identified that can mediate binding and phagocytosis of apoptotic cells by the recognition of PS on these cells such as Tim1, Tim4 and Stabilin-2 (Kobayashi et al., 2007; Park et al., 2008). In addition, there are several bridging molecules such as the plasma proteins: lactadherin, Gas-6 and protein S, that have been described to bind to PS and direct PS to receptors on phagocytes, α_*v*_β_3/5_ integrins and receptors of the TAM receptor family and mediate clearance of PS-positive cells (Raymond et al., 2009). Of these receptors, at least Axl, Tim4 and Stabilin-2 are expressed in red pulp macrophages (Burger and van Bruggen, unpublished) (Figure 4). Thus, this opens the possibility that PS-exposing RBC are cleared in the spleen by phagocytosis by one or more of these PS or PS/ligand receptor pairs. As was already mentioned above, the loss of phospholipid asymmetry and the subsequent exposure of PS on the RBC surface in apoptotic cells may be a general trigger for RBC removal. It also seems that upon RBC storage the susceptibility to stress-induced PS exposure increases, thereby causing a considerable fraction of the RBC to be susceptible to removal after transfusion (Bosman et al., 2011).

Of interest, N-Acetyl-L-Cysteine (NAC) prolong the half-life of circulating mouse erythrocytes *in vivo* RBC drawn from mice that were subsequently treated with NAC exhibited a significantly higher survival rate after the intravenous injection into the sibling mice than those RBC without an NAC treatment (Ghashghaeinia et al., 2012).

## CD47; a molecular switch for RBC phagocytosis

The immunoreceptor signal regulatory protein alpha (SIRPα), expressed by macrophages, is well-known for its ability to inhibit phagocytosis of CD47 expressing cells (Oldenborg et al., 2000; Ishikawa-Sekigami et al., 2006). The CD47-SIRPα interaction (Figure 4) provides a strong negative signal for phagocytosis and can function as a marker of “self” on RBC. Thus, a low level of opsonization might suffice to trigger phagocytosis of a foreign particle that does not express CD47, whereas a “self” particle, such as an RBC, would not be phagocytosed by macrophages in the spleen due to the presence of CD47 on the RBC and the resulting inhibitory signals generated upon contact with macrophage SIRPα. Interestingly, mild hemolytic anemia is seen in Rh-null or protein 4.2-deficient human individuals (Miller et al., 1987; Bruce et al., 2002), which both have strongly reduced RBC CD47 expression levels. Thus it is tempting to speculate that the hemolytic anemia in these individuals may in part be the result of reduced CD47-SIRPα inhibitory signaling to splenic macrophages, possibly in combination with the altered morphology and rheological properties of the RBC in these syndromes.

Based on this, it is of interest to determine whether a reduced expression of CD47 during RBC senescence is also involved in facilitating the uptake of these cells by macrophages. Some studies suggest that there is evidence that point in this direction. One group has found, that a fraction of older murine RBC (>30 days old) show about 20% reduced CD47 expression as compared to a fraction of younger RBC (Fossati-Jimack et al., 2002). Also in another study, RBC storage, which is known to be associated with accelerated RBC clearance following transfusion, was shown to result in loss of CD47 (Anniss and Sparrow, 2002), although we were not able to reproduce these results for RBC stored under standard Dutch blood bank conditions (Burger and van Bruggen, unpublished). These observations, in combination with the previous findings, suggest that clearance of senescent RBC may be regulated by the net result of total signaling through macrophage pro-phagocytic and inhibitory receptors.

However, in 2012 our group has shown that CD47 does not only function as a “don't eat me” signal, but can also act as an “eat me” signal (Burger et al., 2012). In particular, a subset of old RBC present in whole blood was shown to bind and to be phagocytosed via CD47-SIRPα interactions. Furthermore, our group provided evidence that experimental aging of RBC induces a conformational change in CD47 that switches the molecule from an inhibitory into an activating one. Pre-incubation of experimentally aged RBC with human serum prior to the binding assay was required for this activation. In the same study we also demonstrated that aged RBC have the capacity to bind the CD47-binding partner thrombospondin-1 (TSP-1) and that treatment of aged RBC with a TSP-1 derived particle enabled their phagocytosis by human red pulp macrophages. Finally, CD47 on RBC that had been stored for prolonged time was shown to undergo a conformational change and bind TSP-1. These findings reveal a more complex role for CD47-SIRPα interactions in RBC clearance, with CD47 acting as a molecular switch controlling phagocytosis. In addition, we were able to determine that this CD47/SIRPα pathway leading to phagocytosis of RBC is operational in human red pulp macrophages.

## RBC complement receptor 1: mediator in immune-adherence clearance through macrophages

Immune adherence was first described by Nelson (1953) as the immunological reaction between RBC and complement opsonized pathogens. Humans and other higher primates are unique for immune-adherence clearance (IAC) using complement receptor 1 (CR1) on the RBC membrane, which is a critical protection in host defense against blood-borne pathogens such as bacteria. For the vast majority of lower primates, not RBC, but platelets are responsible for binding and transporting circulating complement opsonized particles.

CR1 on human RBC binds complement opsonized particles bearing C3b/C4b in the circulation (Ross and Medof, 1985; Wilson et al., 1987). The opsonized particles are subsequently transported to the spleen and liver where they are removed by residential macrophages, without phagocytosing the RBC. Another group has shown in an *in vitro* study that the transfer rate of immune complexes to monocytes is accelerated once the immune complexes are bound to RBC CR1 compared to unbound opsonized immune complexes, thereby increasing the efficiency of immune complex removal (Emlen et al., 1992). Furthermore, in a mouse transgenic model, where human CR1 is expressed on murine RBC, immune adherence was shown to enhance the resistance of the host to infection with *S. pneumoniae* (Li et al., 2010a).

Recently, interesting work by Melhorn and colleagues on the interplay between RBC and phagocytes in transfer of opsonized particles has been published. It was shown that signal transduction downstream of CR1 after particle binding results in alterations in RBC membrane deformability and in clustering of CR1 on the RBC surface, which enhances binding of the opsonized particle. But more importantly, proof was provided that CR1 ligation leads to ATP secretion, which has a direct stimulatory effect on particle uptake by phagocytes (Melhorn et al., 2013). Thus, the RBC seems to play an active role in the capture of opsonized particles as well as the subsequent transfer of these particles to phagocytes.

## RBC macrophage interactions: future directions

Macrophages play a pivotal role in RBC production, maintenance and clearance. Although it is clear that macrophage–RBC interactions are critical in these processes, the molecular mechanisms behind many of these interactions are still elusive. The role of macrophages in the formation of erythroblastic islands is the most extensively studied process in which RBC macrophage interactions are important. The other two biological phenomena, maintenance and clearance of RBC are less well-understood. Especially, the process in which macrophages of the spleen aid the removal of inclusions in the RBC, are obscure. In the near future efforts should be made to fully understand this process as well as RBC clearance. New techniques such as intravital microscopy might be used to study these processes in detail, thereby generating knowledge that may aid to prevent unwanted RBC destruction in diseases such as β-Thalassemia or hemolytic anemia, or reduce the loss of stored donor RBC after transfusion.

### Conflict of interest statement

The authors declare that the research was conducted in the absence of any commercial or financial relationships that could be construed as a potential conflict of interest.

## References

[B1] Allen T. D. Testa N. G. (1991). Cellular interactions in erythroblastic islands in long-term bone marrow cultures, as studied by time-lapse video. Blood Cells 17, 29–38 2018858

[B2] An X. Mohandas N. (2011). Erythroblastic islands, terminal erythroid differentiation and reticulocyte maturation. Int. J. Hematol. 93, 139–143 10.1007/s12185-011-0779-x 21293952

[B3] Angelillo-Scherrer A. Burnier L. Lambrechts D. Fish R. J. Tjwa M. Plaisance S. (2008). Role of Gas6 in erythropoiesis and anemia in mice. J. Clin. Invest. 118, 583–596 10.1172/JCI30375 18188450 PMC2176185

[B4] Anniss A. M. Sparrow R. L. (2002). Expression of CD47 (integrin-associated protein) decreases on red blood cells during storage. Transfus. Apher. Sci. 27, 233–238 10.1016/S1473-0502(02)00070-8 12509218

[B5] Arashiki N. Kimata N. Manno S. Mohandas N. Takakuwa Y. (2013). Membrane peroxidation and methemoglobin formation are both necessary for band 3 clustering: mechanistic insights into human erythrocyte senescence. Biochemistry 52, 5760–5769 10.1021/bi400405p 23889086 PMC3914982

[B6] Arese P. Turrini F. Schwarzer E. (2005). Band 3/complement-mediated recognition and removal of normally senescent and pathological human erythrocytes. Cell. Physiol. Biochem. 16, 133–146 10.1159/000089839 16301814

[B98] Bachmann A. Esser C. Petter M. Predehl S. von Kalckreuth V. Schmiedel S. (2009). Absence of erythrocyte sequestration and lack of multicopy gene family expression in Plasmodium falciparum from a splenectomized malaria patient. PLoS ONE 4:e7459 10.1371/journal.pone.0007459 19826486 PMC2758591

[B7] Bessis M. (1958). Erythroblastic island, functional unity of bone marrow. Rev. Hematol. 13, 8–11 13555228

[B8] Bessis M. C. Breton-Gorius J. (1962). Iron metabolism in the bone marrow as seen by electron microscopy: a critical review. Blood 19, 635–663 13868561

[B9] Boas F. E. Forman L. Beutler E. (1998). Phosphatidylserine exposure and red cell viability in red cell aging and in hemolytic anemia. Proc. Natl. Acad. Sci. U.S.A. 95, 3077–3081 10.1073/pnas.95.6.3077 9501218 PMC19697

[B99] Bosman G. J. Cluitmans J. C. Groenen Y. A. Werre J. M. Willekens F. L. Novotny V. M. (2011). Susceptibility to hyperosmotic stress-induced phosphatidylserine exposure increases during red blood cell storage. Transfusion 51, 1072–1078 10.1111/j.1537-2995.2010.02929.x 21077907

[B10] Bruce L. J. Ghosh S. King M. J. Layton D. M. Mawby W. J. Stewart G. W. (2002). Absence of CD47 in protein 4.2-deficient hereditary spherocytosis in man: an interaction between the Rh complex and the band 3 complex. Blood 100, 1878–1885 10.1182/blood-2002-03-0706 12176912

[B100] Buffet P. A. Milon G. Brousse V. Correas J. M. Dousset B. Couvelard A. (2006). *Ex vivo* perfusion of human spleens maintains clearing and processing functions. Blood 107, 3745–3752 10.1182/blood-2005-10-4094 16384927

[B11] Burger P. Hilarius-Stokman P. de Korte D. van den Berg T. K. van Bruggen R. (2012). CD47 functions as a molecular switch for erythrocyte phagocytosis. Blood 119, 5512–5521 10.1182/blood-2011-10-386805 22427202

[B12] Chow A. Huggins M. Ahmed J. Hashimoto D. Lucas D. Kunisaki Y. (2013). CD169(+) macrophages provide a niche promoting erythropoiesis under homeostasis and stress. Nat. Med. 19, 429–436 10.1038/nm.3057 23502962 PMC3983996

[B13] Clarke A. R. Maandag E. R. van Roon M. van der Lugt N. M. van der Valk M. Hooper M. L. (1992). Requirement for a functional Rb-1 gene in murine development. Nature 359, 328–330 10.1038/359328a0 1406937

[B14] Crary S. E. Buchanan G. R. (2009). Vascular complications after splenectomy for hematologic disorders. Blood 114, 2861–2868 10.1182/blood-2009-04-210112 19636061 PMC2756197

[B15] Crocker P. R. Werb Z. Gordon S. Bainton D. F. (1990). Ultrastructural localization of a macrophage-restricted sialic acid binding hemagglutinin, SER, in macrophage-hematopoietic cell clusters. Blood 76, 1131–1138 2205308

[B16] Crosby W. H. (1957). Siderocytes and the spleen. Blood 12, 165–170 13403981

[B17] Crosby W. H. (1959). Normal functions of the spleen relative to red blood cells: a review. Blood 14, 399–408 13638340

[B18] Dai C. Chung I. J. Jiang S. Price J. O. Krantz S. B. (2003). Reduction of cell cycle progression in human erythroid progenitor cells treated with tumour necrosis factor alpha occurs with reduced CDK6 and is partially reversed by CDK6 transduction. Br. J. Haematol. 121, 919–927 10.1046/j.1365-2141.2003.04367.x 12786804

[B19] Dautry-Varsat A. Ciechanover A. Lodish H. F. (1983). pH and the recycling of transferrin during receptor-mediated endocytosis. Proc. Natl. Acad. Sci. U.S.A. 80, 2258–2262 10.1073/pnas.80.8.2258 6300903 PMC393798

[B20] De Maria R. Zeuner A. Eramo A. Domenichelli C. Bonci D. Grignani F. (1999). Negative regulation of erythropoiesis by caspase-mediated cleavage of GATA-1. Nature 401, 489–493 10.1038/46809 10519553

[B101] Emlen W. Carl V. Burdick G. (1992). Mechanism of transfer of immune complexes from red blood cell CR1 to monocytes. Clin. Exp. Immunol. 89, 8–17 10.1111/j.1365-2249.1992.tb06869.x 1385769 PMC1554399

[B21] Fabriek B. O. Polfliet M. M. Vloet R. P. van der Schors R. C. Ligtenberg A. J. Weaver L. K. (2007). The macrophage CD163 surface glycoprotein is an erythroblast adhesion receptor. Blood 109, 5223–5229 10.1182/blood-2006-08-036467 17353345

[B22] Fernandez-Boyanapalli R. F. Frasch S. C. McPhillips K. Vandivier R. W. Harry B. L. Riches D. W. (2009). Impaired apoptotic cell clearance in CGD due to altered macrophage programming is reversed by phosphatidylserine-dependent production of IL-4. Blood 113, 2047–2055 10.1182/blood-2008-05-160564 18952895 PMC2651016

[B23] Flores-Figueroa E. Gutierrez-Espindola G. Montesinos J. J. Arana-Trejo R. M. Mayani H. (2002). *In vitro* characterization of hematopoietic microenvironment cells from patients with myelodysplastic syndrome. Leuk. Res. 26, 677–686 10.1016/S0145-2126(01)00193-X 12008086

[B24] Fossati-Jimack L. Azeredo da S. S. Moll T. Kina T. Kuypers F. A. Oldenborg P. A. (2002). Selective increase of autoimmune epitope expression on aged erythrocytes in mice: implications in anti-erythrocyte autoimmune responses. J. Autoimmun. 18, 17–25 10.1006/jaut.2001.0563 11869043

[B25] Franco R. S. (2009). The measurement and importance of red cell survival. Am. J. Hematol. 84, 109–114 10.1002/ajh.21298 19025796

[B26] Ghashghaeinia M. Cluitmans J. C. Akel A. Dreischer P. Toulany M. Koberle M. (2012). The impact of erythrocyte age on eryptosis. Br. J. Haematol. 157, 606–614 10.1111/j.1365-2141.2012.09100.x 22429222

[B27] Hanayama R. Tanaka M. Miwa K. Shinohara A. Iwamatsu A. Nagata S. (2002). Identification of a factor that links apoptotic cells to phagocytes. Nature 417, 182–187 10.1038/417182a 12000961

[B28] Hanspal M. Hanspal J. S. (1994). The association of erythroblasts with macrophages promotes erythroid proliferation and maturation: a 30-kD heparin-binding protein is involved in this contact. Blood 84, 3494–3504 7949103

[B29] Hanspal M. Smockova Y. Uong Q. (1998). Molecular identification and functional characterization of a novel protein that mediates the attachment of erythroblasts to macrophages. Blood 92, 2940–2950 9763581

[B30] Harley J. D. (1965). Role of reduced glutathione in human erythrocytes. Nature 206, 1054–1055 10.1038/2061054a0 5320267

[B31] Hentze M. W. Muckenthaler M. U. Galy B. Camaschella C. (2010). Two to tango: regulation of mammalian iron metabolism. Cell 142, 24–38 10.1016/j.cell.2010.06.028 20603012

[B32] Hornig R. Lutz H. U. (2000). Band 3 protein clustering on human erythrocytes promotes binding of naturally occurring anti-band 3 and anti-spectrin antibodies. Exp. Gerontol. 35, 1025–1044 10.1016/S0531-5565(00)00126-1 11121688

[B33] Iavarone A. King E. R. Dai X. M. Leone G. Stanley E. R. Lasorella A. (2004). Retinoblastoma promotes definitive erythropoiesis by repressing Id2 in fetal liver macrophages. Nature 432, 1040–1045 10.1038/nature03068 15616565

[B34] Inada T. Iwama A. Sakano S. Ohno M. Sawada K. Suda T. (1997). Selective expression of the receptor tyrosine kinase, HTK, on human erythroid progenitor cells. Blood 89, 2757–2765 9108393

[B35] Ishikawa-Sekigami T. Kaneko Y. Okazawa H. Tomizawa T. Okajo J. Saito Y. (2006). SHPS-1 promotes the survival of circulating erythrocytes through inhibition of phagocytosis by splenic macrophages. Blood 107, 341–348 10.1182/blood-2005-05-1896 16141346

[B36] Jacks T. Fazeli A. Schmitt E. M. Bronson R. T. Goodell M. A. Weinberg R. A. (1992). Effects of an Rb mutation in the mouse. Nature 359, 295–300 10.1038/359295a0 1406933

[B37] Ji P. Murata-Hori M. Lodish H. F. (2011). Formation of mammalian erythrocytes: chromatin condensation and enucleation. Trends Cell Biol. 21, 409–415 10.1016/j.tcb.2011.04.003 21592797 PMC3134284

[B38] Kawane K. Fukuyama H. Kondoh G. Takeda J. Ohsawa Y. Uchiyama Y. (2001). Requirement of DNase II for definitive erythropoiesis in the mouse fetal liver. Science 292, 1546–1549 10.1126/science.292.5521.1546 11375492

[B39] Kay M. (2005). Immunoregulation of cellular life span. Ann. N.Y. Acad. Sci. 1057, 85–111 10.1196/annals.1356.005 16399889

[B40] Kay M. M. (2004). Band 3 and its alterations in health and disease. Cell. Mol. Biol. 50, 117–138 15095783

[B41] Kobayashi N. Karisola P. Pena-Cruz V. Dorfman D. M. Jinushi M. Umetsu S. E. (2007). TIM-1 and TIM-4 glycoproteins bind phosphatidylserine and mediate uptake of apoptotic cells. Immunity 27, 927–940 10.1016/j.immuni.2007.11.011 18082433 PMC2757006

[B42] Koury M. J. Bondurant M. C. (1990). Erythropoietin retards DNA breakdown and prevents programmed death in erythroid progenitor cells. Science 248, 378–381 10.1126/science.2326648 2326648

[B43] Kristiansen M. Graversen J. H. Jacobsen C. Sonne O. Hoffman H. J. Law S. K. (2001). Identification of the haemoglobin scavenger receptor. Nature 409, 198–201 10.1038/35051594 11196644

[B44] Kusakabe M. Hasegawa K. Hamada M. Nakamura M. Ohsumi T. Suzuki H. (2011). c-Maf plays a crucial role for the definitive erythropoiesis that accompanies erythroblastic island formation in the fetal liver. Blood 118, 1374–1385 10.1182/blood-2010-08-300400 21628412

[B45] Lang K. S. Lang P. A. Bauer C. Duranton C. Wieder T. Huber S. M. (2005). Mechanisms of suicidal erythrocyte death. Cell. Physiol. Biochem. 15, 195–202 10.1159/000086406 15956782

[B46] Lee E. Y. Chang C. Y. Hu N. Wang Y. C. Lai C. C. Herrup K. (1992). Mice deficient for Rb are nonviable and show defects in neurogenesis and haematopoiesis. Nature 359, 288–294 10.1038/359288a0 1406932

[B47] Lee G. Lo A. Short S. A. Mankelow T. J. Spring F. Parsons S. F. (2006). Targeted gene deletion demonstrates that the cell adhesion molecule ICAM-4 is critical for erythroblastic island formation. Blood 108, 2064–2071 10.1182/blood-2006-03-006759 16690966 PMC1895542

[B48] Lee G. Spring F. A. Parsons S. F. Mankelow T. J. Peters L. L. Koury M. J. (2003). Novel secreted isoform of adhesion molecule ICAM-4: potential regulator of membrane-associated ICAM-4 interactions. Blood 101, 1790–1797 10.1182/blood-2002-08-2529 12406883

[B49] Lee J. C. Gimm J. A. Lo A. J. Koury M. J. Krauss S. W. Mohandas N. (2004). Mechanism of protein sorting during erythroblast enucleation: role of cytoskeletal connectivity. Blood 103, 1912–1919 10.1182/blood-2003-03-0928 14563645

[B50] Lee S. H. Crocker P. R. Westaby S. Key N. Mason D. Y. Gordon S. (1988). Isolation and immunocytochemical characterization of human bone marrow stromal macrophages in hemopoietic clusters. J. Exp. Med. 168, 1193–1198 10.1084/jem.168.3.1193 3049905 PMC2189021

[B51] Leimberg M. J. Prus E. Konijn A. M. Fibach E. (2008). Macrophages function as a ferritin iron source for cultured human erythroid precursors. J. Cell. Biochem. 103, 1211–1218 10.1002/jcb.21499 17902167

[B52] Li J. Wang J. P. Ghiran I. Cerny A. Szalai A. J. Briles D. E. (2010a). Complement receptor 1 expression on mouse erythrocytes mediates clearance of Streptococcus pneumoniae by immune adherence. Infect. Immun. 78, 3129–3135 10.1128/IAI.01263-09 20439480 PMC2897369

[B53] Li L. Fang C. J. Ryan J. C. Niemi E. C. Lebron J. A. Bjorkman P. J. (2010b). Binding and uptake of H-ferritin are mediated by human transferrin receptor-1. Proc. Natl. Acad. Sci. U.S.A. 107, 3505–3510 10.1073/pnas.0913192107 20133674 PMC2840523

[B54] Li MO. Sarkisian M. R. Mehal W. Z. Rakic P. Flavell R. A. (2003). Phosphatidylserine receptor is required for clearance of apoptotic cells. Science 302, 1560–1563 10.1126/science.1087621 14645847

[B55] Liu X. S. Li X. H. Wang Y. Shu R. Z. Wang L. Lu S. Y. (2007). Disruption of palladin leads to defects in definitive erythropoiesis by interfering with erythroblastic island formation in mouse fetal liver. Blood 110, 870–876 10.1182/blood-2007-01-068528 17431131

[B56] Low P. S. (1986). Structure and function of the cytoplasmic domain of band 3: center of erythrocyte membrane-peripheral protein interactions. Biochim. Biophys. Acta 864, 145–167 10.1016/0304-4157(86)90009-2 2943319

[B58] Lutz H. U. (2004). Innate immune and non-immune mediators of erythrocyte clearance. Cell Mol. Biol. 50, 107–116 15095782

[B57] Lutz H. U. Nater M. Stammler P. (1993). Naturally occurring anti-band 3 antibodies have a unique affinity for C3. Immunology 80, 191–196 8262548 PMC1422201

[B59] Lutz H. U. Stammler P. Fasler S. (1990). How naturally occurring anti-band 3 antibodies stimulate C3b deposition to senescent and oxidatively stressed red blood cells. Biomed. Biochim. Acta 49, S224–S229 2386510

[B60] Maddala R. Reddy V. N. Epstein D. L. Rao V. (2003). Growth factor induced activation of Rho and Rac GTPases and actin cytoskeletal reorganization in human lens epithelial cells. Mol. Vis. 9, 329–336 12876554

[B61] Mannu F. Arese P. Cappellini M. D. Fiorelli G. Cappadoro M. Giribaldi G. (1995). Role of hemichrome binding to erythrocyte membrane in the generation of band-3 alterations in beta-thalassemia intermedia erythrocytes. Blood 86, 2014–2020 7655029

[B62] Manwani D. Bieker J. J. (2008). The erythroblastic island. Curr. Top. Dev. Biol. 82, 23–53 10.1016/S0070-2153(07)00002-6 18282516 PMC3234703

[B63] McGrath K. E. Kingsley P. D. Koniski A. D. Porter R. L. Bushnell T. P. Palis J. (2008). Enucleation of primitive erythroid cells generates a transient population of “pyrenocytes” in the mammalian fetus. Blood 111, 2409–2417 10.1182/blood-2007-08-107581 18032705 PMC2234067

[B64] Means R. T. (2004). Hepcidin and cytokines in anaemia. Hematology 9, 357–362 10.1080/10245330400018540 15763974

[B65] Mebius R. E. Kraal G. (2005). Structure and function of the spleen. Nat. Rev. Immunol. 5, 606–616 10.1038/nri1669 16056254

[B66] Melhorn M. I. Brodsky A. S. Estanislau J. Khoory J. A. Illigens B. Hamachi I. (2013). CR1-mediated ATP release by human red blood cells promotes CR1 clustering and modulates the immune-transfer process. J. Biol. Chem. 288, 31139–31153 10.1074/jbc.M113.486035 24022490 PMC3829426

[B67] Miller Y. E. Daniels G. L. Jones C. Palmer D. K. (1987). Identification of a cell-surface antigen produced by a gene on human chromosome 3 (cen-q22) and not expressed by rhnull cells. Am. J. Hum. Genet. 41, 1061–1070 3120581 PMC1684367

[B68] Mohandas N. Prenant M. (1978). Three-dimensional model of bone marrow. Blood 51, 633–643 630113

[B69] Morelli A. E. Larregina A. T. Shufesky W. J. Zahorchak A. F. Logar A. J. Papworth G. D. (2003). Internalization of circulating apoptotic cells by splenic marginal zone dendritic cells: dependence on complement receptors and effect on cytokine production. Blood 101, 611–620 10.1182/blood-2002-06-1769 12393562

[B102] Munasinghe A. Ileperuma M. Premawansa G. Handunnetti S. Premawansa S. (2009). Spleen modulation of cytoadherence properties of Plasmodium falciparum. Scand. J. Infect. Dis. 41, 538–539 10.1080/00365540902971195 19449257

[B70] Muta K. Krantz S. B. Bondurant M. C. Dai C. H. (1995). Stem cell factor retards differentiation of normal human erythroid progenitor cells while stimulating proliferation. Blood 86, 572–580 7541668

[B71] Nelson R. A. Jr. (1953). The immune-adherence phenomenon; an immunologically specific reaction between microorganisms and erythrocytes leading to enhanced phagocytosis. Science 118, 733–737 10.1126/science.118.3077.733 13122009

[B72] Oldenborg P. A. Zheleznyak A. Fang Y. F. Lagenaur C. F. Gresham H. D. Lindberg F. P. (2000). Role of CD47 as a marker of self on red blood cells. Science 288, 2051–2054 10.1126/science.288.5473.2051 10856220

[B73] Pantaleo A. Giribaldi G. Mannu F. Arese P. Turrini F. (2008). Naturally occurring anti-band 3 antibodies and red blood cell removal under physiological and pathological conditions. Autoimmun. Rev. 7, 457–462 10.1016/j.autrev.2008.03.017 18558362

[B74] Park S. Y. Jung M. Y. Kim H. J. Lee S. J. Kim S. Y. Lee B. H. (2008). Rapid cell corpse clearance by stabilin-2, a membrane phosphatidylserine receptor. Cell Death Differ. 15, 192–201 10.1038/sj.cdd.4402242 17962816

[B75] Piomelli S. Seaman C. (1993). Mechanism of red blood cell aging: relationship of cell density and cell age. Am. J. Hematol. 42, 46–52 10.1002/ajh.2830420110 8416296

[B76] Ramos P. Casu C. Gardenghi S. Breda L. Crielaard B. J. Guy E. (2013). Macrophages support pathological erythropoiesis in polycythemia vera and beta-thalassemia. Nat. Med. 19, 437–445 10.1038/nm.3126 23502961 PMC3618568

[B77] Raymond A. Ensslin M. A. Shur B. D. (2009). SED1/MFG-E8: a bi-motif protein that orchestrates diverse cellular interactions. J. Cell. Biochem. 106, 957–966 10.1002/jcb.22076 19204935 PMC2742659

[B78] Rhodes M. M. Kopsombut P. Bondurant M. C. Price J. O. Koury M. J. (2008). Adherence to macrophages in erythroblastic islands enhances erythroblast proliferation and increases erythrocyte production by a different mechanism than erythropoietin. Blood 111, 1700–1708 10.1182/blood-2007-06-098178 17993612 PMC2214751

[B79] Ribeil J. A. Zermati Y. Vandekerckhove J. Cathelin S. Kersual J. Dussiot M. (2007). Hsp70 regulates erythropoiesis by preventing caspase-3-mediated cleavage of GATA-1. Nature 445, 102–105 10.1038/nature05378 17167422

[B80] Ross G. D. Medof M. E. (1985). Membrane complement receptors specific for bound fragments of C3. Adv. Immunol. 37, 217–267 10.1016/S0065-2776(08)60341-7 3159188

[B81] Sadahira Y. Yoshino T. Monobe Y. (1995). Very late activation antigen 4-vascular cell adhesion molecule 1 interaction is involved in the formation of erythroblastic islands. J. Exp. Med. 181, 411–415 10.1084/jem.181.1.411 7528776 PMC2191848

[B82] Schnitzer B. Sodeman T. Mead M. L. Contacos P. G. (1972). Pitting function of the spleen in malaria: ultrastructural observations. Science 177, 175–177 10.1126/science.177.4044.175 4339353

[B83] Secchiero P. Melloni E. Heikinheimo M. Mannisto S. Di PR. Iacone A. (2004). TRAIL regulates normal erythroid maturation through an ERK-dependent pathway. Blood 103, 517–522 10.1182/blood-2003-06-2137 12969966

[B84] Seki M. Shirasawa H. (1965). Role of the reticular cells during maturation process of the erythroblast. 3. the fate of phagocytized nucleus. Acta Pathol. Jpn. 15, 387–405 5899496 10.1111/j.1440-1827.1965.tb01930.x

[B85] Skutelsky E. Danon D. (1972). On the expulsion of the erythroid nucleus and its phagocytosis. Anat. Rec. 173, 123–126 10.1002/ar.1091730111 5028062

[B86] Soni S. Bala S. Gwynn B. Sahr K. E. Peters L. L. Hanspal M. (2006). Absence of erythroblast macrophage protein (Emp) leads to failure of erythroblast nuclear extrusion. J. Biol. Chem. 281, 20181–20189 10.1074/jbc.M603226200 16707498

[B87] Suenobu S. Takakura N. Inada T. Yamada Y. Yuasa H. Zhang X. Q. (2002). A role of EphB4 receptor and its ligand, ephrin-B2, in erythropoiesis. Biochem. Biophys. Res. Commun. 293, 1124–1131 10.1016/S0006-291X(02)00330-3 12051776

[B88] Tordjman R. Delaire S. Plouet J. Ting S. Gaulard P. Fichelson S. (2001). Erythroblasts are a source of angiogenic factors. Blood 97, 1968–1974 10.1182/blood.V97.7.1968 11264160

[B89] Ulyanova T. Jiang Y. Padilla S. Nakamoto B. Papayannopoulou T. (2011). Combinatorial and distinct roles of alpha(5) and alpha(4) integrins in stress erythropoiesis in mice. Blood 117, 975–985 10.1182/blood-2010-05-283218 20956802 PMC3035084

[B90] Wilkins B. S. Wright D. H. (2000). Illustrated Pathology of the Spleen. Cambridge: Cambridge University Press 10.1017/CBO9780511545979

[B91] Willekens F. L. Roerdinkholder-Stoelwinder B. Groenen-Dopp Y. A. Bos H. J. Bosman G. J. van den Bos A. G. (2003). Hemoglobin loss from erythrocytes *in vivo* results from spleen-facilitated vesiculation. Blood 101, 747–751 10.1182/blood-2002-02-0500 12393566

[B92] Wilson J. G. Andriopoulos N. A. Fearon D. T. (1987). CR1 and the cell membrane proteins that bind C3 and C4. A basic and clinical review. Immunol. Res. 6, 192–209 10.1007/BF02918091 2960763

[B93] Yokoyama T. Etoh T. Kitagawa H. Tsukahara S. Kannan Y. (2003). Migration of erythroblastic islands toward the sinusoid as erythroid maturation proceeds in rat bone marrow. J. Vet. Med. Sci. 65, 449–452 10.1292/jvms.65.449 12736425

[B94] Yokoyama T. Kitagawa H. Takeuchi T. Tsukahara S. Kannan Y. (2002). No apoptotic cell death of erythroid cells of erythroblastic islands in bone marrow of healthy rats. J. Vet. Med. Sci. 64, 913–919 10.1292/jvms.64.913 12419868

[B95] Yoshida H. Kawane K. Koike M. Mori Y. Uchiyama Y. Nagata S. (2005). Phosphatidylserine-dependent engulfment by macrophages of nuclei from erythroid precursor cells. Nature 437, 754–758 10.1038/nature03964 16193055

[B96] Zamai L. Secchiero P. Pierpaoli S. Bassini A. Papa S. Alnemri E. S. (2000). TNF-related apoptosis-inducing ligand (TRAIL) as a negative regulator of normal human erythropoiesis. Blood 95, 3716–3724 10845902

[B97] Zermati Y. Fichelson S. Valensi F. Freyssinier J. M. Rouyer-Fessard P. Cramer E. (2000). Transforming growth factor inhibits erythropoiesis by blocking proliferation and accelerating differentiation of erythroid progenitors. Exp. Hematol. 28, 885–894 10.1016/S0301-472X(00)00488-4 10989189

[B98a] Zhang D. Kiyatkin A. Bolin J. T. Low P. S. (2000). Crystallographic structure and functional interpretation of the cytoplasmic domain of erythrocyte membrane band 3. Blood 96, 2925–2933 11049968

